# Iterative Sequencing and Variant Screening (ISVS) as a novel pathogenic mutations search strategy - application for *TMPRSS3* mutations screen

**DOI:** 10.1038/s41598-017-02315-w

**Published:** 2017-05-31

**Authors:** Urszula Lechowicz, Tomasz Gambin, Agnieszka Pollak, Anna Podgorska, Piotr Stawinski, Andre Franke, Britt-Sabina Petersen, Malgorzata Firczuk, Monika Oldak, Henryk Skarzynski, Rafal Ploski

**Affiliations:** 1 Department of Genetics, Institute of Physiology and Pathology of Hearing, Warsaw, Poland; 2 Institute of Computer Science, Warsaw University of Technology, Warsaw, Poland; 3 Department of Medical Genetics, Institute of Mother and Child at Warsaw, Warsaw, Poland; 4 Institute of Clinical Molecular Biology, Kiel University, Kiel, Germany; 5 Department of Immunology, Center of Biostructure Research, Medical University of Warsaw, Warsaw, Poland; 6 Oto-Rhino-Laryngology Surgery Clinic, Institute of Physiology and Pathology of Hearing, Warsaw, Poland; 7 Department of Medical Genetics, Center of Biostructure, Medical University of Warsaw, Warsaw, Poland

## Abstract

Autosomal recessive diseases (ARD) are typically caused by a limited number of mutations whose identification is challenged by their low prevalence. Our purpose was to develop a novel approach allowing an efficient search for mutations causing ARD and evaluation of their pathogenicity without a control group. We developed Iterative Sequencing and Variant Screening (ISVS) approach based on iterative cycles of gene sequencing and mutation screening, and *ISVS Simulator* software (http://zsibio.ii.pw.edu.pl/shiny/isvs/) for assessment of detected variants’ significance. As shown by simulations, ISVS efficiently identifies and correctly classifies pathogenic mutations except for cases where the gene of interest has extremely high number of low frequency nonpathogenic variants. By applying ISVS, we found 4 known and 9 novel (p.C73Y, p.S124L, p.C194Mfs*17, c.782 + 2 T > A, c.953-5 A > G, p.L325Q, p.D334Mfs*24, p.R436G, p.M448T) *TMPRSS3* variants among deaf patients. For 3 known and 5 novel variants the disease association was supported by *ISVS Simulator* odds >90:1. Pathogenicity of 6 novel mutations has been supported by *in-silico* predictions of variants’ deleteriousness. By directly comparing variant prevalence in patients and controls, disease association was demonstrated only for two variants and it was relatively weak (P < 0.05). Summarizing, ISVS strategy and *ISVS Simulator* are useful for detection of genetic variants causing AR diseases.

## Introduction

Autosomal recessive diseases (ARD) are often caused by a limited number of mutations. Accurate knowledge of pathogenic mutation profile in a given population is important as it helps to interpret results of diagnostic tests in the patients and may greatly facilitate design of efficient screening strategies. Whereas numerous *in silico* tools for mutation effect prediction are available they are not perfect and the assignment of pathogenicity remains a challenge, especially for variants with very low population frequency^1^. For such rare variants the association with disease defined as more frequent occurrence of a variant among the patients than controls is an important *sine qua non* criterion of pathogenicity^2^. However, the formal demonstration of an association may be difficult as it requires multiple patients with given mutation and a control group of appropriate size.

The purpose of our work was to develop and validate a novel approach allowing: (i) an efficient search for mutations causing ARD, (ii) evaluation of their pathogenicity by testing for disease association without using a control group.

The proposed mutation search strategy is based on iterative cycles of gene sequencing combined with focused mutation screening, which we termed Iterative Sequencing and Variant Screening (ISVS). Testing for association with disease relies on the analysis of statistical significance (using simulations) of co-occurrence among the patients of a given sequence variant with other rare potentially pathogenic variants in the same gene.

The developed approach was validated by searching for pathogenic variants in the *TMPRSS3* gene in a cohort of 2247 subjects with sensorineural hearing loss (SNHL). We found 13 different rare *TMPRSS3* variants, nine of which were novel. ISVS simulations showed for six of these variants (4 novel) strong (>1000:1) evidence for disease association, for two (1 novel) the evidence was moderate (90:1) and for five (4 novel) - weak.

## Subjects and Methods

### Principle of Iterative Sequencing and Variant Screening (ISVS)

ISVS is initiated by minimum one known pathogenic mutation (Mut. 1) which is screened for by a focused test (for example Real Time PCR – RT PCR) in the whole cohort of patients. Next, all the found samples heterozygous for the Mut. 1 are subjected to sequencing of all coding regions of gene (*GENE*) which is likely to reveal, in a number of cases, other variants either known as pathogenic or possibly pathogenic according to available criteria (database frequency, functional annotations, etc). Next, all these variants enter the second round of screening in the whole cohort of patients, similar as in the first round, and then *GENE* sequencing is similarly repeated. The procedure is iteratively continued until no potentially interesting variants are detected by sequencing.

### *In silico* ISVS analysis

To assess the performance of ISVS method we first performed experiments *in silico* modelling search for pathogenic *GENE* mutations among patients with *disease*. We modelled the parameters used in this analysis according to the experiment performed later on using real samples and aimed at detecting *TMPRSS3* mutations in approx. 2000 subjects with SNHL. In particular, we assumed that the prevalence of disease (f1) in the general population is 0.1%, as 1 to 2 in 1000 newborn children in developed countries are born with SNHL^3^. The frequency of cases affected by bi-allelic variants in *GENE* (f2) was assumed as 2% of all cases - an estimate based on the frequency of *TMPRSS3* causative mutations among SNHL patients of different ethnicity^4–10^. Therefore, the frequency of individuals with *TMPRSS3* homozygous or compound heterozygous variants in the general population f3 = f1*f2 = 0.002%.

In a single experiment, the genotypes were generated for the cohort of *N* = 2000 individuals. For the given number *m* of distinct pathogenic mutations, the allele frequency spectrum was sampled from the beta distribution with parameters *alpha* = 0.5, *beta* = 10 and then scaled to ensure that cumulative allele frequency of all mutations equals to *f3*. Parameters of beta distribution were tuned based on the real allele frequency spectrum observed in the cohort described in this manuscript. Next, for each mutation the status of maternal and paternal alleles for all individuals has been randomly assigned to 0 or 1 using allele probabilities obtained in the previous step. Individuals with homozygous or compound heterozygous variants were labeled as affected due to mutations in *GENE* and (N*f1 - N*f3) subjects were marked as additional group of patients with deafness caused by other genetic or non-genetic factors.

In addition to pathogenic mutations, for every patient we sampled the zygosity status of *k* non-pathogenic variants assuming the cumulative frequency of these variants of 0.05. The rationale for choosing 0.05 was based on analysis of ExAC^11^ data which showed that the cumulative rare allele frequency (<0.01) of missense mutations in the *TMPRSS3* gene was 0.027. It can be safely assumed that in majority of populations pathogenic variants with prevalence >0.01 are likely to be known and thus we can safely reject any variant with frequency >0.01 during ISVS process. Since some rare variants are not reported in ExAC during testing variant pathogenicity by *in silico* ISVS simulation we arbitrarily increased the value to 0.05. As discussed further on, assuming higher cumulative frequency of non-pathogenic variants is conservative as the performance of ISVS decreases with increase of this parameter.

For such a cohort of all affected individuals (with or without *GENE* mutations) we performed an ISVS simulation, which works as follows. First, we randomly select a single individual with homozygous or compound heterozygous pathogenic *GENE* mutation. Next, remaining affected subjects are screened for the mutations found in the first individual. All carriers of these mutations are then checked for additional variants across the whole gene (which corresponds to sequencing part in the real experiment). New individuals with homozygous and compound heterozygous variants are added to the list of patients in whom disease is potentially caused by mutations in *GENE*. Then the screening is performed again using the list of novel variants and it is followed by the sequencing. This procedure is repeated until there are no novel variants to be screened in the next step. Finally, we summarize the results of experiments by calculating the fraction of identified variants out of all *m* mutations considered in the experiment.

### Testing variant pathogenicity by *in silico* ISVS simulation

To cause a recessive condition a pathogenic variant on one allele has to co-occur with another pathogenic variant on the other allele. Thus, in patients with recessive diseases causative variants are likely to co-occur (*in-trans* configuration) with other pathogenic variants but this is not the case for rare variants with no phenotypic effect. We tested if this information can be used to discriminate pathogenic and non-pathogenic variants identified in ISVS experiment using the following procedure.

First, the set of ISVS simulations (i.e. *in silico* repetitions of the ISVS experiment) is randomly split into the training (80% of simulations) and testing part (20% of simulations). Next, using the data from training simulations we build a classification model. For each variant identified in the ISVS experiment we calculate two numbers: (i) the number of individuals for which this variant occurs *in-trans* with any other variant identified by ISVS procedure, and (ii) the total number of occurrences of this variant in the analyzed cohort. These two features along with the predefined class label for the variant (i.e. pathogenic/non-pathogenic) are then used to train Support Vector Machine (SVM) classifier^12^. Performance of other classification methods and details of SVM model tuning are described in supplementary material. Subsequently, the classification model is used to predict the class of variants from the set of test simulations. Finally, to calculate the algorithm performance we repeat above steps under 5-fold cross validation. In addition, to provide a single “pathogenicity” score for an individual variant we also calculate the likelihood ratio, i.e. the probability of variant being pathogenic vs non-pathogenic.

### Development of a web application to *in silico* simulate ISVS experiments

We have developed a software which performs a series of *in-silico* simulations of ISVS experiment (publicly available at http://zsibio.ii.pw.edu.pl/shiny/isvs/). Application is implemented in R programming language (https://cran.r-project.org/) using shiny framework and rCharts library (https://ramnathv.github.io/rCharts/) to create user interface. To speed up the computations these simulations are run on the server in parallel using 30 cores on our local cluster.

### Application of ISVS for screening for *TMPRSS3* mutations among patients with hearing loss (HI)

The patients were selected from the Polish SNHL subjects consulted in the Genetic Department of the Institute of Physiology and Pathology of Hearing (IPPH) between 2000 and 2014. Genomic DNA (gDNA) was extracted from the peripheral blood of all patients using salting out method. The hearing loss was determined by pure-tone audiometry at 500 Hz, 1 kHz, 2 kHz, 4 kHz and 8 kHz and was at least 20 dBHL. The group included 2247 (1204 females and 1043 males) unrelated SNHL patients from IPPH. The mean level of HI was moderate (i.e. 41~70 dBHL) at 500 kHz, 1 kHz, 2 kHz and severe (i.e. 71~95 dBHL) at 4 kHz, 8 kHz. The mean age of SNHL onset was 12 years. We excluded from this study patients who already had established cause of HI, that is those with syndromic SNHL of known genetic origin, and also carriers of two mutations in connexin genes *GJB2* and/or *GJB6* (recessive inheritance). *GJB2/GJB6* linked deafness is relatively frequent in Caucasians accounting for up to 20% of nonsydromic cases^13, 14^. Whereas different pathogenic mutations may co-occur by chance it remains a rare event^3, 15^, thus the exclusion of patients with already defined cause of SNHL can be expected to increase the yield of the search for mutations in any gene(s) not tested previously.

Written informed consent was obtained from all the subjects or parents/guardians of participating children and all procedures were approved by the bioethical commission at Institute of Physiology and Pathology of Hearing, Warsaw, Poland.

Screening of the background Polish population was performed using DNA samples from anonymous unrelated individuals who underwent paternity testing at Department of Forensic Medicine, Medical University of Warsaw, all these subjects gave written informed consent for anonymous use of their DNA for research.

Naming of all *TMPRSS3* mutations is in accordance with the recommendations of Human Genome Variation Society (HGVS). NM_024022.2 was applied as the reference *TMPRSS3* sequence for cDNA, while at the protein level NP_076927.1 sequence was used.

### Whole Exome Sequencing (WES)

One female proband with congenital, profound SNHL from a family suggestive of recessive type of inheritance (healthy parents and a sister with SNHL) with excluded common *GJB2* mutations and NC_012920.1:m.1555 A > G, NC_012920.1:m.3243A>G was selected for WES. WES was performed at the ICMB in Kiel on HiSeq2000 platform with 2 × 100 bp reads using TruSeq Exome Enrichment Kit (Illumina). The data were analyzed as previously described^16^.

### Laboratory steps of ISVS based *TMPRSS3* screening among SNHL patients

ISVS was initiated by WES in a single proband which revealed two frameshift variants NM_024022.2:c.[208delC(;)579dupA]. These two variants were screened for by RT PCR in the whole cohort of patients with SNHL. Next, all samples heterozygous for any of the two variants underwent Sanger sequencing of the *TMPRSS3* gene which revealed, in 20 cases, variants either known as pathogenic or tentatively classified by us as such. All these variants were then screened for by RT PCR among the cohort of SNHL patients similar as in the former round and then Sanger sequencing was similarly repeated. The procedure was continued until no potentially interesting variants were detected by sequencing.

The tentative assignment of pathogenicity (and therefore inclusion in RT PCR screening) was based on previous reports and/or prevalence <1% in 1000Genomes or CG69^17^ ESPSP6500^18^ and ExAC^11^ databases. Furthermore, the prevalence of each variant was determined in a cohort of 597 samples from background population of Poland using RT PCR. Detected variants with prevalence in the abovementioned cohorts <1% were regarded as ‘potentially pathogenic’.

Screening with RT PCR was performed using Assay on Demand reagents (Life Technologies, Carlsbad, CA, USA). Sanger sequencing was performed with ABI PRISM 3500XL capillary sequencer (Life Technologies) and covered all coding exons and boundary intronic sequences of *TMPRSS3* (sequences of primers used are shown in Supplementary Table 1 (Supp. Table 1).

**Table 1:** Prevalence of rare variants in the *TMPRSS3* gene detected among Polish HI patients and controls.

Variant	het	hom	PATIENTS					CONTROLS			P value
			allele mut	allele sum	freq. %	het	hom	allele mut	allele sum	freq. %	
p.H70Tfs*19	20	3	26	4376	0.59	0	0	0	1058	0	0.012
p.A138E	17	2	21	4604	0.46	2	0	2	1918	0.1	0.029
p.M448T^	10	0	10	4596	0.22	0	0	0	1222	0	0.13
p.S124L^	5	0	5	4524	0.11	0	0	0	1046	0	0.59
p.C194Mfs*17^	4	0	4	4594	0.09	2	0	2	1932	0.1	1.0
c.953-5 A > G^	4	0	4	4560	0.09	0	0	0	1058	0	1.0
p.R109W	2	0	2	4506	0.04	0	0	0	998	0	1.0
p.D334Mfs*24^	2	0	2	4618	0.04	0	0	0	1080	0	1.0
p.C73Y^^^	1	0	1	4562	0.02	0	0	0	1046	0	1.0
c.782 + 2 T > A^	1	0	1	4206	0.02	0	0	0	1042	0	1.0
p.L325Q^	1	0	1	4566	0.02	0	0	0	1018	0	1.0
p.A426T	1	0	1	4104	0.02	0	0	0	1112	0	1.0
p.R436G^	1	0	1	4604	0.02	0	0	0	986	0	1.0

^Mutation not reported to date; het- heterozygous, hom- homozygous, allele mut- number of mutated alleles, allele sum - number of tested alleles.

### Testing of potentially pathogenic *TMPRSS3* variants for association with HI

All potentially pathogenic *TMPRSS3* variants were tested for association with SNHL using two approaches: (i) prevalence of each variant was compared among SNHL patients and controls by Fisher’s exact test using SPSS 11.5 K (SPSS Inc., Chicago, USA), and *p* values of less than 0.05 were considered statistically significant, (ii) among SNHL patients in whom given variant was found in at least one chromosome, the prevalence of additional potentially pathogenic rare variants (present presumably in the other chromosome) was compared with distribution obtained from ISVS simulations.

### RNA expression study

For probands and parents with p.M448T mutation total RNA was isolated from peripheral blood using of MagNA Pure Compact RNA Isolation Kit (Roche, Basel, Switzerland) using MagNA Pure Compact Instrument. cDNA synthesis was performed with the Maxima First Strand cDNA Synthesis Kits for RT-qPCR (ThermoFisherScientific, Waltham, USA).

### Bioinformatics analysis of mutations’ effects

The probable deleterious effects of the found missense variants were predicted using SIFT (http://sift.jcvi.org)^19^, MetaSVM^1^, MutationTaster2^20^ and PolyPhen-2^21^. TMPRSS3 model was generated with M4T server^22^, using the structure of the homologous transmembrane serine protease hepsin as a model (PDB code 1Z8G). The protein graphics was produced with the PyMOL Molecular Graphics System, Version 1.3, Schrödinger, LLC. Domain conservation consensus and TMPRSS3 sequence alignments were retrieved from Simple Modular Architecture Research Tool (SMART) database^23^.

Novel *TMPRSS3* splice site variants were analyzed using the Alamut Visual Software 2.8 (Interactive Biosoftware, Rouen, France).

## Results

### In silico modelling of ISVS search for TMPRSS mutations in SNHL patients

By performing 10,000 ISVS simulations with parameters adjusted for *TMPRSS* gene and SNHL we found that, on average, by 4.75 steps of an ISVS experiment the success rate (i.e. the fraction of experiments where all affected individuals were detected) was 0.98. Average classification accuracy of pathogenic vs. non-pathogenic variants was 0.97 (at confidence 0.5) or 0.83 (at confidence 0.95). When SVM classification was used the latter value raised to 0.85.

### ISVS Simulator software

In order to facilitate the more broad use of ISVS we developed software (ISVS Simulator) that enables user to test the performance of the method under scenarios different than the considered by us *TMPRSS* mutation screen in SNHL subjects. In particular, the application allows user to modify various parameters, such as disease cohort size, disease prevalence, fraction of disease cases explained by mutation in the given gene, expected numbers of distinct pathogenic and non-pathogenic mutations in the gene, cumulative frequency of non-pathogenic variants, parameters of beta distribution for variant allele frequencies and the number of simulations to be repeated in the single experiment.

**Figure 1:**
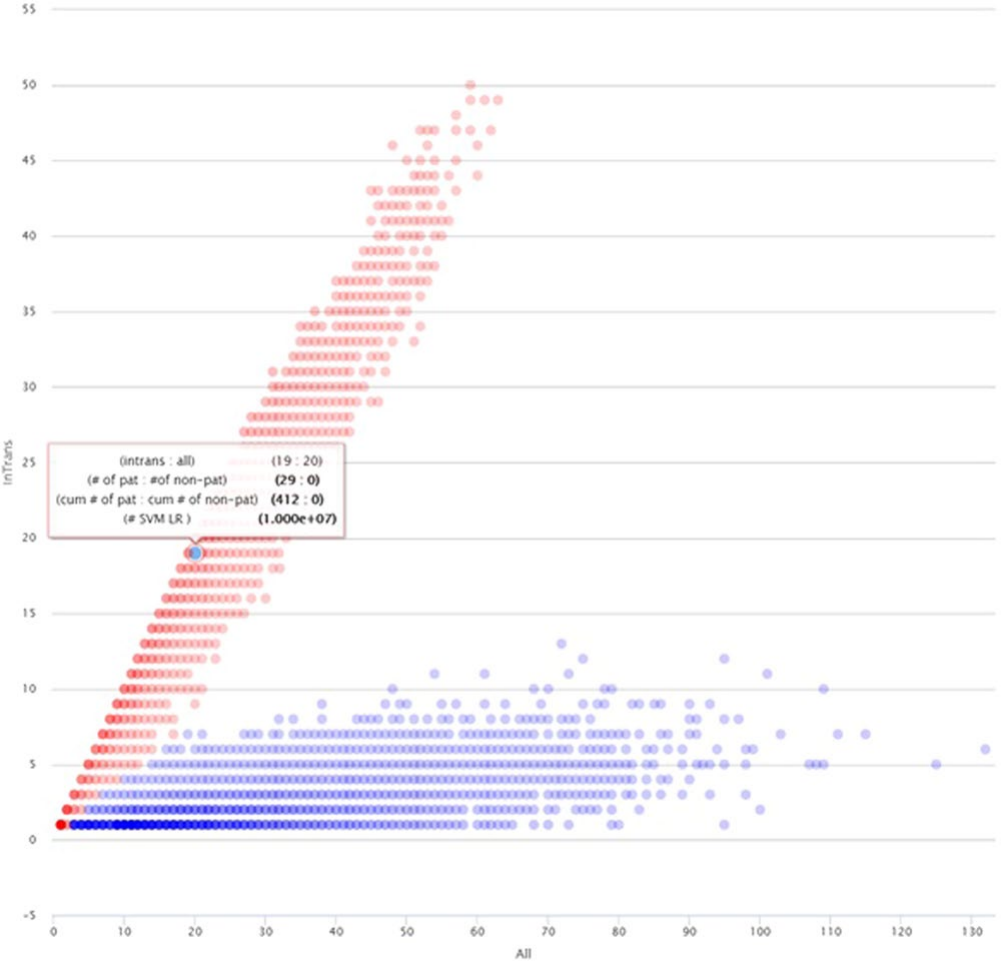
Screen-shot from ISVS simulator showing an example of “Fraction bi-allelic plot” obtained using default settings. Note information about given (x,y) point provided in the tooltip. This particular point indicates that if in a real ISVS experiment given variant was found in 20 patients of whom 19 also had a rare variant in the other allele (intrans:all = 19:20) there is considerable evidence (#SVM LR = 107) that the variant is pathogenic.

The experiment results are presented in three tabs, located on the right side of the web page. First one, the “Fraction bi-allelic plot” presents the number of bi-allelic (*in-trans*) events associated with the given mutation (y-axis) in reference to the total number occurrences of this mutation (x-axis, Fig. 1). Every point of this plot represents a set of mutations that were observed in exactly x individuals where in y cases ISVS identified another mutation on the second allele. The tooltip associated with each point plot displays information about: (i) the x, y values (intrans: all); (ii) the total number of pathogenic and non-pathogenic mutations which yielded given (x, y) in ISVS simulations (# of pat: # of non-pat); (iii) cumulative value of the latter (cum # of pat: cum # of non-pat). This was calculated according to principle that if given (x, y) were observed only for pathogenic mutations and the same was seen for (x, y-1), (x, y-2), etc., than for the (x, y) point the cumulative number of pathogenic is conservative to sum the “# of pat” values with the value for (x, y-1), (x, y-2), etc. point(s); (iv) SVM likelihood ratio, i.e. probability that mutation is pathogenic/probability that mutation is non-pathogenic. The intensities of red and blue colors correspond to the number of observed pathogenic and non-pathogenic variants, respectively. A part of the plot can be enlarged by marking it with the mouse.

The “ISVS summary” tab shows a summary statistics including: an average number of samples for which the targeted gene had to be sequenced, an average number of identified samples with bi-allelic pathogenic variants, an average number of pathogenic mutation carriers, and ISVS success rate, i.e. the fraction of simulations in which all individuals with pathogenic bi-allelic mutations were detected. In addition, the classification accuracies are reported for 0.5 and 0.95 confidence levels. Detailed classification results (including numbers of TP (true positive), TN (true negative) and classification accuracies) for confidence levels varying between 0.5 and 1 are also presented in the “Classification results” tab.

### *In silico* analysis of the performance of ISVS

To study the performance and robustness of ISVS we repeated the above-mentioned experiment using different set of input parameters. To ensure the stability of results, for a given set of parameters ISVS simulation were repeated 10,000 times. We collected the following metrics: (i) **successRate** – the fraction of ISVS simulations in which all individuals affected by bi-allelic pathogenic mutation were detected (ii) **patFractDetected** – the average fraction of patients with bi-allelic pathogenic mutation who were properly identified; (iii) **mutFractDetected** – the average fraction of pathogenic variants that were properly identified; (iv) **avgSteps** – the average number of steps in ISVS experiment; (v) **sequencedSampleNr** – the average number of sequenced samples; (vi) **screenedVarsNr** – the average number of screened variants.

The aim of our analysis was to evaluate how the above-mentioned metrics depend on the change of the selected input parameters, including: disease cohort size (default = 2000), frequency of disease individuals within the population (default = 0.001), the fraction of disease individuals affected by bi-allelic variants in the GENE (default = 0.02), the number of distinct pathogenic mutations (default = 10), the number of distinct non-pathogenic (default = 20) and the cumulative frequency of non-pathogenic mutations (default = 0.05). Complete results from this analysis are presented in Supplementary Figures S1–3 (Supp. Fig. S1–3).

We observed that first three performance metrics (i.e. **successRate**, **patFractDetected** and **mutFractDetected**) all achieve very high (>0.9) values for most of the configurations. Smaller **successRate** (<0.9) were observed only when the disease cohort size was smaller than 1000 or the fraction of individuals with bi-allelic variants in disease cohort was smaller than 1%. This shows the limitations of ISVS method in terms of the minimal number of patients who need to be ascertained in order to effectively identify pathogenic variants and those subjects in whom these variants cause the disease.

Another finding is that the average number of ISVS steps (**avgSteps)** is limited never exceeding five in our simulations, regardless of the input parameters. We observed that the number of steps further decreases when the fraction of affected individuals by bi-allelic variants (or cumulative frequency of pathogenic mutations) grows. This is consistent with our expectations, since higher cumulative frequency of pathogenic variants enlarges the number of variant occurrences that “link” subsequent sequencing and screening steps in ISVS experiment.

We found that ISVS performance decreases with the increase of number and cumulative frequency of non-pathogenic variants. In particular, the increase of number and cumulative frequency of non-pathogenic variants causes sharp increase in the amount of variants which need to be screened and samples which need to be sequenced. Even more importantly, high numbers and high cumulative frequency of nonpathogenic variants decrease the accuracy of SVM classification. Sensitivity of classification is predominantly affected by high cumulative frequency of non-pathogenic variants whereas specificity appears especially sensitive to presence of high number of low frequency non-pathogenic mutations. (Supp. Fig. S3). These results indicate that ISVS is less useful for long genes which are likely to have relatively high numbers and high cumulative frequency of rare non-pathogenic variants.

We also studied the performance of ISVS method when the disease cohort is a mixture of two ethnically different populations. We assumed that the same set of pathogenic and non-pathogenic variants is present in both groups but the allele frequencies of those variants differ between populations. In such a setting, we analyzed the performance of ISVS for various proportions of two populations sizes, i.e. from 1:0, 0.9:0.1, …, up to 1:1. As expected we observed a small decrease in the average fraction of detected pathogenic mutations or the average fraction of properly identified affected individuals, when one population is much larger than the other. However, when the sizes of both populations become more balanced, the performance of ISVS returns to the level observed for single ethnically homogenous population.

### *TMPRSS3* mutations in Polish SNHL population

**Figure 2:**
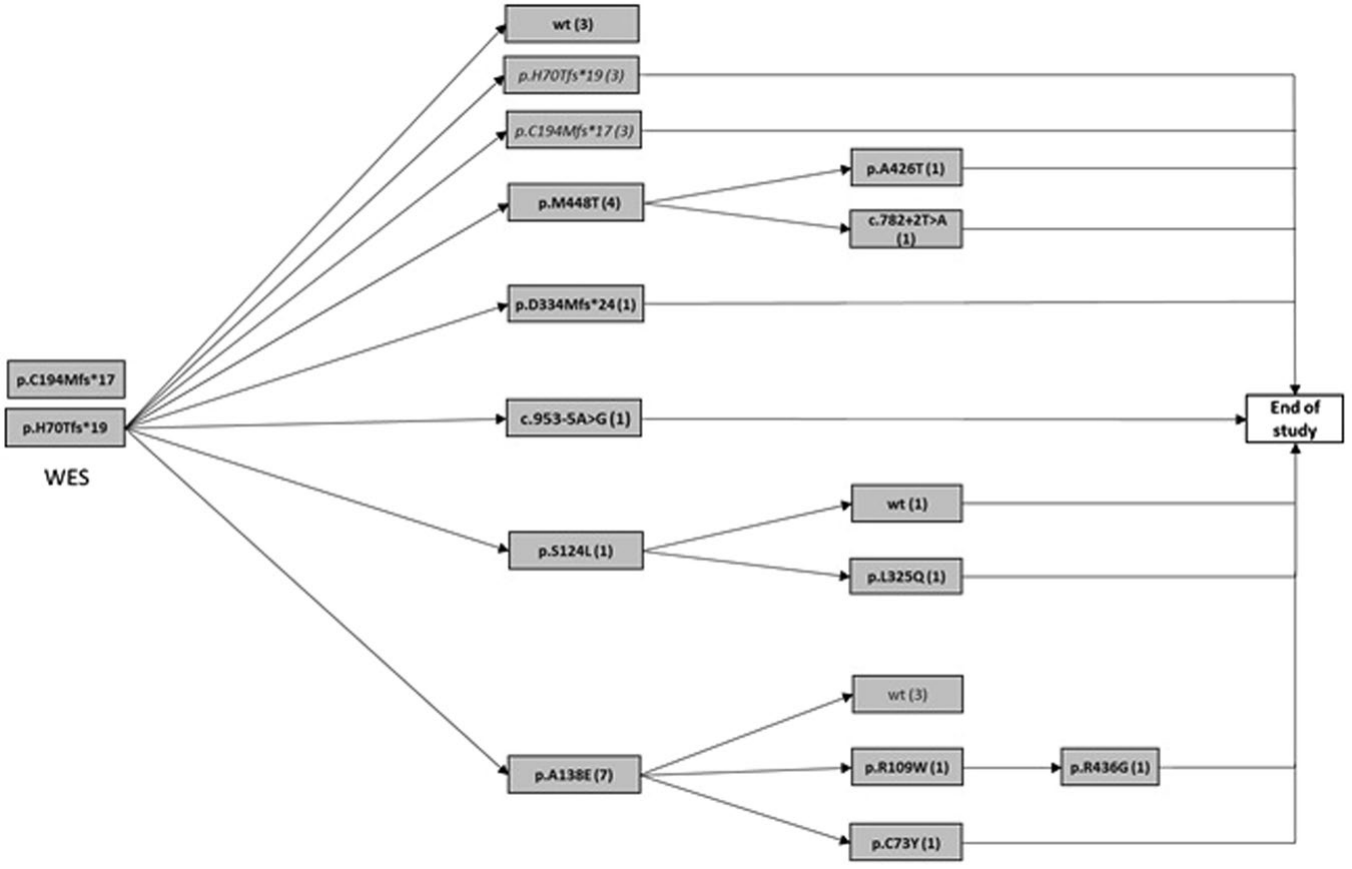
Summary of findings during cascade *TMPRSS* mutation screening by ISVS in a cohort of HI subjects. WES – whole exome sequencing; arrows – cycles of Real Time PCR screening; filled boxes - variants found by Sanger sequencing, (number of samples with each variant is given in parentheses); italics – variants observed at a previous stage.

The practical performance of ISVS procedure as applied to *TMPRSS3* mutation screening among subjects with SNHL is shown in Fig. 2 and the summary of potentially pathogenic variants found is shown in Table 1. We found 4 previously described as well as 9 novel variants. *TMPRSS3* variants which were not reported to date are: p.C73Y, p.S124L, p.C194Mfs*17, c.782 + 2 T > A, c.953-5 A > G, p.L325Q, p.D334Mfs*24, p.R436G, p.M448T whereas the known variants include p.H70Tfs*19, p.R109W, p.A138E, p.A426T. Sanger chromatograms showing novel variants are presented in Supp. Fig. S4.

In the control population the aforementioned variants were either absent or had low allelic frequency (0.1% for p.A138E and p.C194Mfs*17) (Table 1).

In Table 2 we show frequency of second *TMPRSS3* alteration among patients with rare variants in this gene. According to *ISVS Simulator*, for two known mutations (p.H70Tfs*19, p.A138E) as well as four novel variants (p.M448T, p.S124L, p.C194Mfs*17, c.953-5 A > G) the odds for pathogenicity exceeded 1000:1. For another two variants p.D334Mfs*24 (novel) and p.R109W the odds were ~90:1. It should be noted that only two of the abovementioned variants with pathogenicity supported by *ISVS Simulator* were associated with SNHL when their prevalence was compared with population controls (p.H70Tfs*19, p.A138E, Table 1).

**Table 2:** Statistical assessment of the disease-association of the detected *TMPRSS3* mutations using *ISVS Simulator*.

Mutation	BAM/all	#pat/#non-pat		SVM LR	Second mutations found in patients
		Observed	Cumulat.		
p.H70Tfs*19	20/23	92/0	929/0	10^7^	p.A138E (7), p.M448T (4), p.H70Tfs*19 (3), p.C194Mfs*17 (3), wt (3), p.S124L (1), c.953-5 A > G (1), p.D334Mfs*24 (1)
p.A138E	16/19	349/0	1076/0	10^7^	p.H70Tfs*19 (7), wt (3), p.A138E (2), p.C73Y (1), p.R109W (1), p.S124L (1), p.C194Mfs*17 (1), c.953-5 A > G (1), p.D334Mfs*24 (1), p.M448T (1)
p.M448T^	10/10	361/0	2599/0	10^7^	p.H70Tfs*19 (4), c.953-5 A > G (2), p.S124L (1), p.A138E (1), c.782 + 2 T > A (1), p.A426T (1),
p.S124L^	4/5	1689/0	1689/0	1390	p.H70Tfs*19 (1), p.A138E (1), p. L325Q (1), p.M448T (1), wt (1)
p.C194Mfs*17^	4/4	2204/1	2204/1	7126	p.H70Tfs*19 (3), p.A138E (1)
c.953-5 A > G^	4/4	2204/1	2204/1	7126	p.M448T (2), p.H70Tfs*19 (1), p.A138E (1)
p.R109W	2/2	4709/57 = 82	4709/57 = 82	90	p.A138E (1), p.R436G (1)
p.D334Mfs*24^	2/2	4709/57 = 82	4709/57 = 82	90	p.H70Tfs*19 (1), p.A138E (1)
p.L325Q^	1/1	7429/655 = 11	7429/655 = 11	10	p.S124L (1)
p.R436G^	1/1	7429/655 = 11	7429/655 = 11	10	p.R109W (1)
p.A426T	1/1	7429/655 = 11	7429/655 = 11	10	p.M448T (1)
c.782 + 2 T > A^	1/1	7429/655 = 11	7429/655 = 11	10	p.M448T (1)
p.C73Y^	1/1	7429/655 = 11	7429/655 = 11	10	p.A138E (1)

BAM/all– number with bi-allelic mutations/total number with given mutation. in brackets - number of patients with a given mutation. The settings for *ISVS Simulator*: number of patients = 2 200; disease prevalence 1/1000; fraction of disease cases explained by mutation in the gene: 0.02; Number of iterations: 10 000; Number of pathogenic mutations: 10; Number of non-pathogenic mutations: 20; Cumulative frequency of non-pathogenic variants: 0.05.

### Prediction of biological effect of novel *TMPRSS3* variants

*Frameshift or splice-site variants* The c.579dupA and c.999delC mutations are predicted to cause frameshift and premature termination of protein (p.C194Mfs*17 and p.D334Mfs*24, respectively). The c.782 + 2 T > A and c.953-5 A > G mutations are likely to affect splicing of *TMPRSS3* mRNA since both in minor and major class introns at the + 2 position (second position at the 5′ end of an intron) T is always present whereas at position -5 (the 5^th^ base from the 3’ end of an intron) C, T or A but not G occur^24^. Both mutations are predicted as most probably affecting splicing variants (Table 3).

**Table 3:** Splice site mutation predictions with the usage of Alamut bioinformatics algorithms. SSF- SpliceSiteFinder-like; HSF- Human Splicing Finder; – no data.

Variant	Alamut bioinformatics algorithms					Interpretation
	SSF	MaxEntScan	NNSPLICE	GeneSplicer	HSF	
c.782 + 2 T > A	100%	100%	100%	100%	100%	Broken WT Donor Site, most probably affecting splicing
c.953-5A > G	—	61%	—	46%	<1%	Broken WT Acceptor Site, most probably affecting splicing

*Missense variants* Three out of five missenses are predicted to be deleterious/disease-causing by all five prediction tools used (SIFT, PolyPhen-2, MutationTaster2, MetaSVM and MetaLR, Table 4); p.S124L was predicted as benign by all the tools, p.M448T was “diseases causing” only according to MutationTaster2. In addition to the use of the abovementioned prediction programs we also evaluated properties of each variant by protein modelling.

**Table 4:** Bioinformatics analysis of novel missense variants in *TMPRSS3* gene.

TMPRSS3 mutation	SIFT	PolyPhen-2	MutationTaster2	MetaSVM	MetaLR
p.C73Y	damaging	probably damaging	disease causing	damaging	damaging
p.S124L	tolerated	benign	polymorphism	tolerated	tolerated
p.L325Q	damaging	probably damaging	disease causing	damaging	damaging
p.R436G	damaging	probably damaging	disease causing	damaging	damaging
p.M448T	tolerated	benign	disease causing	tolerated	tolerated

*p.C73Y* (*c.218* *G* > *A*) The LDLRA domain contains two loops interconnected by three disulfide bonds, formed by highly conserved Cys residues. The conservation of amino acids in TMPRSS3 LDLR domain is presented in Supp. Fig. S5A. According to the predicted topology model, C73 forms a disulfide bond with C85, establishing the domain scaffold (InterPro, ref: *doi: 10.1093/nar/gku1243*). Mutation of C73 to Y disables the putative disulfide bond formation and is likely to affect conformation of the domain and may cause protein destabilization (Supp. Fig. S5D).

*p.S124L* (*c.371* *C* > *T*) S124 is located in the SRCR domain of TMPRSS3. A polar residue is conserved at this position what may suggest that the residue is involved in the stabilization of the adjacent loop comprising amino acids 162-170, and therefore may be involved in structural stabilization of the domain (Supp. Fig. S5B,D).

*p.L325Q* (*c.974* *T* > *A*) *and p.R436G* (*c.1306* *C* > *G*) Both mutations are placed in the proteolytic domain but are located in a substantial distance from the active site (Supp. Fig. S5C,D). L325 is a highly conserved, hydrophobic amino acid, buried inside the protein structure. Substitution of Leu into a charged Gln may negatively affect the stability of this domain. R436 is located at a protein surface and its side chain is exposed to solvent. A positively charged residue is conserved at this position, suggesting a putative role in the domain structural stability.

*p.M448T* (*c.1343* *T* > *C*) The novel p.M448T (c.1343 T > C) mutation is located close to the splice site. Since in homologous proteins there are variants of Ile or Thr at this position we speculated that this variant may affect mRNA amount rather than the protein structure. In order to check this we performed direct sequencing of *TMPRSS3* cDNA and DNA encompassing the p.M448T in a sample isolated from peripheral blood of heterozygous subjects. cDNA sequencing revealed lower dose of p.M448T allele vs. the wild type allele, in contrast of gDNA sequencing showed equal doses of both alleles (Supp. Fig. S6). This suggests that p.M448T may have a deleterious effect on splicing and/or mRNA stability.

## Discussion

Here we describe ISVS - a novel strategy for detection of bi-allelic pathogenic mutations in a large cohort of patients affected by autosomal recessive disease. ISVS takes advantage of the fact that patients with an autosomal recessive disease who carry a pathogenic variant in a single chromosome should have another pathogenic variant (in the other chromosome). ISVS consists of cyclic rounds of sequencing of the exons/splice sites of a gene combined with focused screening of patients’ cohort for all variants with likely pathogenicity found at the sequencing step. This strategy allows a cost efficient search for ultra-rare, potentially pathogenic variants of a gene implicated in any AR disease. ISVS is particularly effective if a given gene accounts only for minority of cases (due to locus heterogeneity and/or relatively many cases being caused by polygenic or non-genetic factors). Early onset non syndromic hearing loss (NSHL), blindness or mental retardation are examples of prevalent diseases where ISVS can be applied. Importantly, through simulations (using freely available *ISVS Simulator* software) it is possible to test statistical significance of the associations between the found variants and the disease without referring to a control group. We demonstrated that our simple (two-feature based) score has a good discriminative power and can be used as a new predictor of variant pathogenicity. Although including additional features, such as functional annotation scores, mutation frequencies across populations, could potentially increase classification performance, we decided not to use it in order to keep the classification model simple and allow easy use in combination with other independent pathogenicity predictors without the risk of any hidden circularity. As discussed by Grimm *et al*.^25^, the most valuable prediction scores are those that are unrelated and orthogonal to other existing methods.

Using *ISVS Simulator* we assessed the method’s performance contingent on a number of population/mutation parameters. The simulations revealed that within wide range of tested parameters ISVS is highly accurate, i.e. identifies majority (>90%) of pathogenic mutations present in the disease cohort. Moreover, the number of steps in ISVS is usually limited (i.e. up to five steps), regardless of selected input data. We also showed that ISVS works well when a disease cohort is a mixture of two ethnically diverse populations, although a large disproportion in the cohort sizes may have negative effect on the overall method’s performance.

We found that the ISVS performance decreases significantly with the increase of number and cumulative frequency of non-pathogenic variants. In particular, there is an increase in number of samples required to be sequenced, increase in number of variants selected for screening and decrease of the accuracy of SVM classification of pathogenic vs nonpathogenic variants. These results indicate that ISVS is less useful for very long genes which are likely to have relatively high numbers and high cumulative frequency of rare non-pathogenic variants. However, this limitation does not preclude generally high ISVS usefulness due to the fact that majority of genes are relatively short: the coding sequence of 95% of all human genes is shorter than 4400 bp with the median length (1302 bp) similar to the length of the *TMPRSS3* gene (1365 bp) which we used to validate ISVS in practice^11^.

The general knowledge of ISVS performance together with possibility to perform *in silico* under different scenarios should help to decide if performing any particular ISVS experiment would be cost effective in comparison to alternative approaches, e.g. direct sequencing of entire cohort.

To further validate ISVS, we applied it for screening of a cohort of Polish NSHL patients with the aim to identify and characterize pathogenic *TMPRSS3* mutations in this population. *TMPRSS3* screening in NSHL is suitable for ISVS for the following reasons: (i) NSHL has diverse causes but in a significant proportion of cases it is due to genetic defects with AR inheritance. (ii) AR NSHL displays pronounced locus heterogeneity with 85 genes/loci listed in OMIM (http://omim.org/phenotypicSeries/PS220290), (iii) the *TMPRSS3* gene mutations are a relatively rare cause of NSHL in Caucasians^4^, with so far no reports from Polish population. Furthermore, as mentioned previously the length of the *TMPRSS3* gene is typical for majority of human genes.

By the ISVS approach we found four known (p.H70Tfs*19, p.R109W, p.A138E, p.A426T) and nine novel *TMPRSS3* variants: p.C73Y, p.S124L, p.C194Mfs*17, c.782 + 2 T > A, c.953-5 A > G, p.L325Q, p.D334Mfs*24, p.R436G, p.M448T. For three of the known variants (p.H70Tfs*19, p.R109W, p.A138E) and five of the novel variants (p.S124L, p.C194Mfs*17, c.953-5 A > G, p.D334Mfs*24, p.M448T) the disease association was supported by *ISVS Simulator* with odds of 90:1 or more.

It should be emphasized that by directly comparing variant prevalence in patients with controls, despite sizable numbers of subjects in both cohorts, the evidence for disease association was obtained only for two variants (p.H70Tfs*19, p.A138E, previously known) and it was relatively weak (P < 0.05).

Our results concerning six of the novel mutations have been validated by prediction of mutations’ effects. The variants p.C194Mfs*17, c.782 + 2 T > A, c.953-5 A > G, p.D334Mfs*24 are expected to cause protein truncation due to splice site mutation or frameshift. Both *in silico* and cDNA analyses suggest that p.M448T affects splicing rather than acts as a missense mutation (interestingly, p.M448T was the 3^rd^ most prevalent mutation in our study). The pathogenicity of p.C73Y may result from the destruction of the C73-C89 disulfide bond, which is likely to affect the structure of LDLRA domain of the TMPRSS3 protein. An overview of the location of novel TMPRSS3 variants relative to protein domains and the previously described TMPRSS3 mutations is shown in Supp. Fig. S7.

According to our results, the frequency of *TMPRSS3* mutations in Polish SNHL population is at least 1.91% (43/2247), which is close to the prevalence of 1% of childhood NSHL in Caucasians and 2.5% in a Korean cohort^4, 9, 26^. In other studies, among Koreans the prevalence of 5.9 and 8.3% of *TMPRSS3* mutations in total AR NSHL and postlingual AR NSHL patients, respectively, was reported^10^. Similar prevalence was found in Tunisian^27^ and Turkish population^28^.

In conclusion, we propose ISVS as a strategy and *ISVS Simulator* software as a tool for detection of genetic variants causing AR diseases. We validate the ISVS approach providing the *TMPRSS3* mutation profile of Polish SNHL patients.

## Electronic supplementary material

supplementary materials
