# Supplementary material for: Iterative Sequencing and Variant Screening (ISVS) as a novel pathogenic mutations search strategy - application for TMPRSS3 mutations screen
Source: Sci Rep. 2017 May 31;7:2543. doi: 10.1038/s41598-017-02315-w (PMC5451398; doi:10.1038/s41598-017-02315-w)
Supplement: Supplementary file 1 — supplementary materials [file 41598_2017_2315_MOESM1_ESM.pdf]

SUPPLEMENTARY MATERIALS

Title

Iterative Sequencing and Variant Screening (ISVS) as a novel pathogenic mutations search strategy - application for *TMPRSS3* mutations screen

Authors:

Urszula Lechowicz[1], Tomasz Gambin[2,3], Agnieszka Pollak[1], Anna Podgorska[1], Piotr Stawinski[1], Andre Franke[4], Britt-Sabina Petersen[4], Malgorzata Firczuk[5], Monika Oldak[1], Henryk Skarzynski[6], Rafal Ploski[7]*

Affiliation:

[1] Department of Genetics, Institute of Physiology and Pathology of Hearing, Warsaw, Poland
[2] Institute of Computer Science, Warsaw University of Technology, Warsaw, Poland
[3] Department of Medical Genetics, Institute of Mother and Child at Warsaw, Warsaw, Poland
[4] Institute of Clinical Molecular Biology, Kiel University, Kiel, Germany
[5] Department of Immunology, Center of Biostructure Research, Medical University of Warsaw, Warsaw, Poland
[6] Oto-Rhino-Laryngology Surgery Clinic, Institute of Physiology and Pathology of Hearing, Warsaw, Poland,
[7] Department of Medical Genetics, Centre of Biostructure, Medical University of Warsaw

* Corresponding author: Rafal Ploski, Prof., MD, PhD; Department of Medical Genetics, Centre of Biostructure Research, Medical University of Warsaw, Warsaw, Pawinskiego 3C, 02-106, Poland tel.: + 48 22 572 0606, fax: + 48 22 572 0696, e-mail: rploski@wp.pl

Selection of classification method and model tuning

To select the optimal model for classification of variant pathogenicity we compared the performance of five state-of-the-art supervised machine learning methods, including, decision trees (from R package “CART”), linear discriminant analysis (from R package “MASS”), support vector machines (SVM, from R package “kernlab”), random forest (from R package “RF”) penalized logistic regression (from R package “plr”). For SVM we considered models with linear and radial kernels. The classifiers were evaluated on the raw output of ISVS simulator executed with default parameters and 1000 iterations. Performance comparison of algorithms and model tuning was performed using R package “caret”. This algorithms’ evaluation (see Supplementary Table S2) revealed that SVM, Random Forest and Penalized Logistic Regression significantly outperform two other algorithms (Decision Trees and LDA). In the same time, we found no statistically significant differences among three best performing methods (using comparison test implemented in “caret” R package). Moreover, evaluation results for SVM with radial and linear kernels were comparable.

Since there was no obvious winner we decided to use SVM algorithm for classification of variant pathogenicity. In particular, we selected SVM with linear kernel, because it is not recommended practice to use SVM with other (e.g. radial) kernels when observations from different classes are likely to be linearly separable. Finally, we fine-tuned the SVM model to select optimal value for “C” parameter (optimal model was found for C=1, see Supplementary Table S3).

SUPPLEMENTARY TABLES

Supplementary Table S1. Sequences of primers used in Sanger sequencing

**Primers**	
**Name**	**Sequence 5'**
TMPR3e1F	ccgccctctcagagttacag
TMPR3e1R	ttgttttcacctgtcccaca
TMPR3e2F	tgaccaagatgcacctgatg
TMPR3e2R	ccccacagggacagtcagt
TMPR3e3F	ctagagaatgtgccccttgg
TMPR3e3R	taattaaggctgggcagcag
TMPR3e4F	gcactctgaaagagctgttgg
TMPR3e4R	tacagatgggaagggtcagg
TMPR3e5F	cagggatccagagtcactgc
TMPR3e5R	agagcgttaaagcacccaat
TMPR3e6F	ttgccagggtgagtgaactt
TMPR3e6R	tattgggccatactccctca
TMPR3e7F	atctggggcatttttcacag
TMPR3e7R	ctccagcaggtaggggtaca
TMPR3e8F	cccttgcagcacttgtctta
TMPR3e8R	tgatgatgatgggtccacag
TMPR3e9F	ggaccacatcttgcctgataa
TMPR3e9R	aactgatgccaacaccaaca
TMP3e10F	tgctgtgagctgatcgtttt
TMP3e10R	tgactgtgtcccgagcag
TMP3e11F	gcgacacaccagagagcat
TMP3e11R	ttcttctccacgccctgtaa
TMP3e12F	gtcccaactccatagcaagc
TMP3e12R	accaagtcactgctgctgaa
TMP3e13F	agaacagccccacaattcc
TMP3e13R	ctcagagctccaagggtgtc

Supplementary Table S2. Evaluation of performance (accuracy, kappa) of various classification algorithms used for discrimination between pathogenic and nonpathogenic variants

**Accuracy**						
	Min.	1st qu	Median	Mean	3rd qu	Max.
Decision Tree	0.8902	0.8971	0.9322	0.9174	0.9353	0.9385
LDA	0.9159	0.9204	0.9253	0.9239	0.9274	0.9301
SVM_RADIAL	0.9684	0.9698	0.9725	**0.9721**	0.9732	0.9774
SVM_LINEAR	0.9684	0.9696	0.9725	**0.9721**	0.9736	0.9778
Random Forest	0.968	0.9694	0.9729	**0.9719**	0.9736	0.9767
Penalized Logistic Regression	0.9684	0.9696	0.9729	**0.9722**	0.9741	0.9778
**Kappa**						
	Min.	1st qu	Median	Mean	3rd qu	Max.
Decision Tree	0.7758	0.79	0.8628	0.832	0.8691	0.8754
LDA	0.8282	0.8376	0.8476	0.8449	0.852	0.8577
SVM_RADIAL	0.9361	0.9389	0.9445	**0.9436**	0.946	0.9544
SVM_LINEAR	0.9361	0.9386	0.9445	**0.9436**	0.9467	0.9551
Random Forest	0.9354	0.9382	0.9452	**0.9433**	0.9467	0.953
Penalized Logistic Regression	0.9361	0.9386	0.9452	**0.9439**	0.9477	0.9551

Supplementary Table S3. Fine-tuning of the SVM model to select optimal value for "C" parameter

C	Accuracy	Kappa
1.00E-03	0.861986	0.7155854
1.00E-02	0.9401231	0.8782235
**1.00E-01**	**0.9615572**	**0.922119**
1.00E+00	0.9720541	0.9435726
1.00E+01	0.9703162	0.940164

Supplementary Figure S1: Sensitivity of ISVS to input parameters

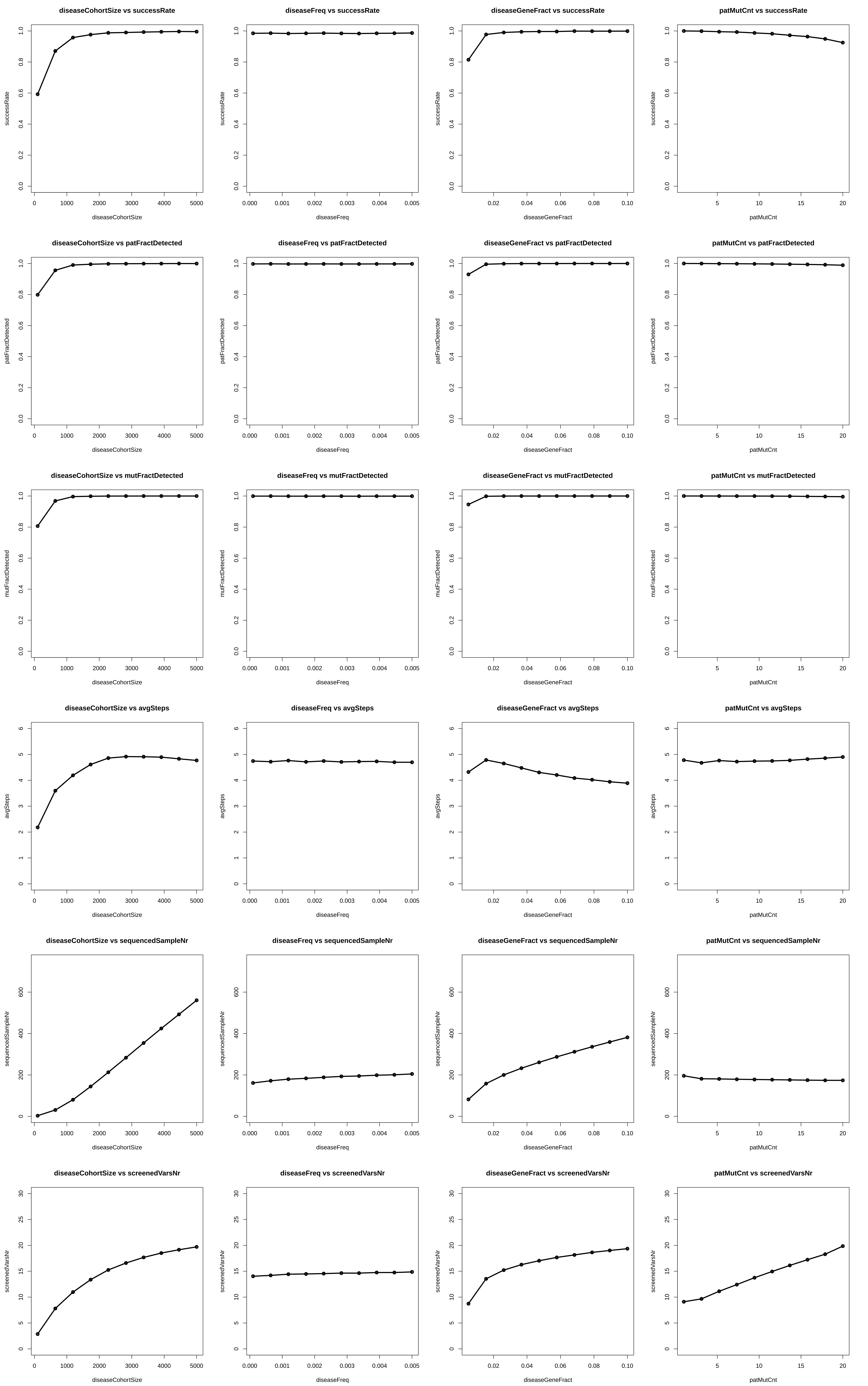

Supplementary Figure S1: Sensitivity of ISVS to input parameters (continue)

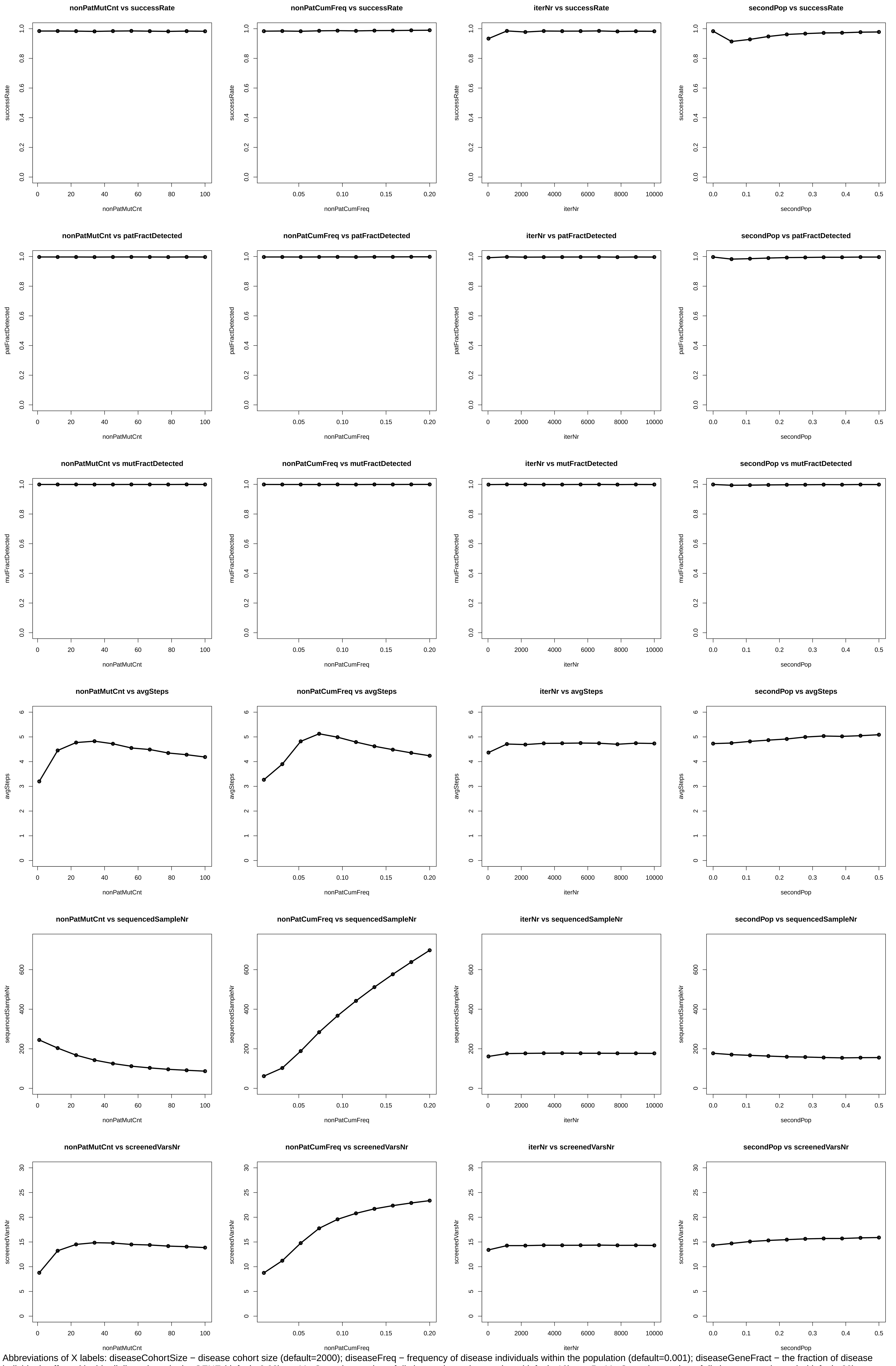

Abbreviations of X labels: diseaseCohortSize − disease cohort size (default=2000); diseaseFreq − frequency of disease individuals within the population (default=0.001); diseaseGeneFract − the fraction of disease individuals affected by bi−allelic variants in the GENE (default=0.02); patMutCnt − the number of distinct pathogenic mutations (default=10); nonPatMutCnt − the number of distinct non−pathogenic (default=20); nonPatCumFreq − cumulative frequency of non−pathogenic mutations (default=0.05); iterNr − number of repetitions of ISVS experiments (default=10000); secondPop − the ratio of population sizes (default=0, i.e. single population)

Abbreviations of Y labels: successRate − the fraction of ISVS simulations in which all individuals affected by bi−allelic pathogenic mutation were detected; patFractDetected − the average fraction of patients with bi−allelic pathogenic mutation who were properly identified; mutFractDetected − the average fraction of pathogenic variants that were properly identified; avgSteps − the average number of steps in ISVS experiment; sequencedSampleNr − the average number of sequenced samples; screenedVarsNr − the average number of screened variants

Supplementary Figure S2: Sensitivity of variant classification to input parameters

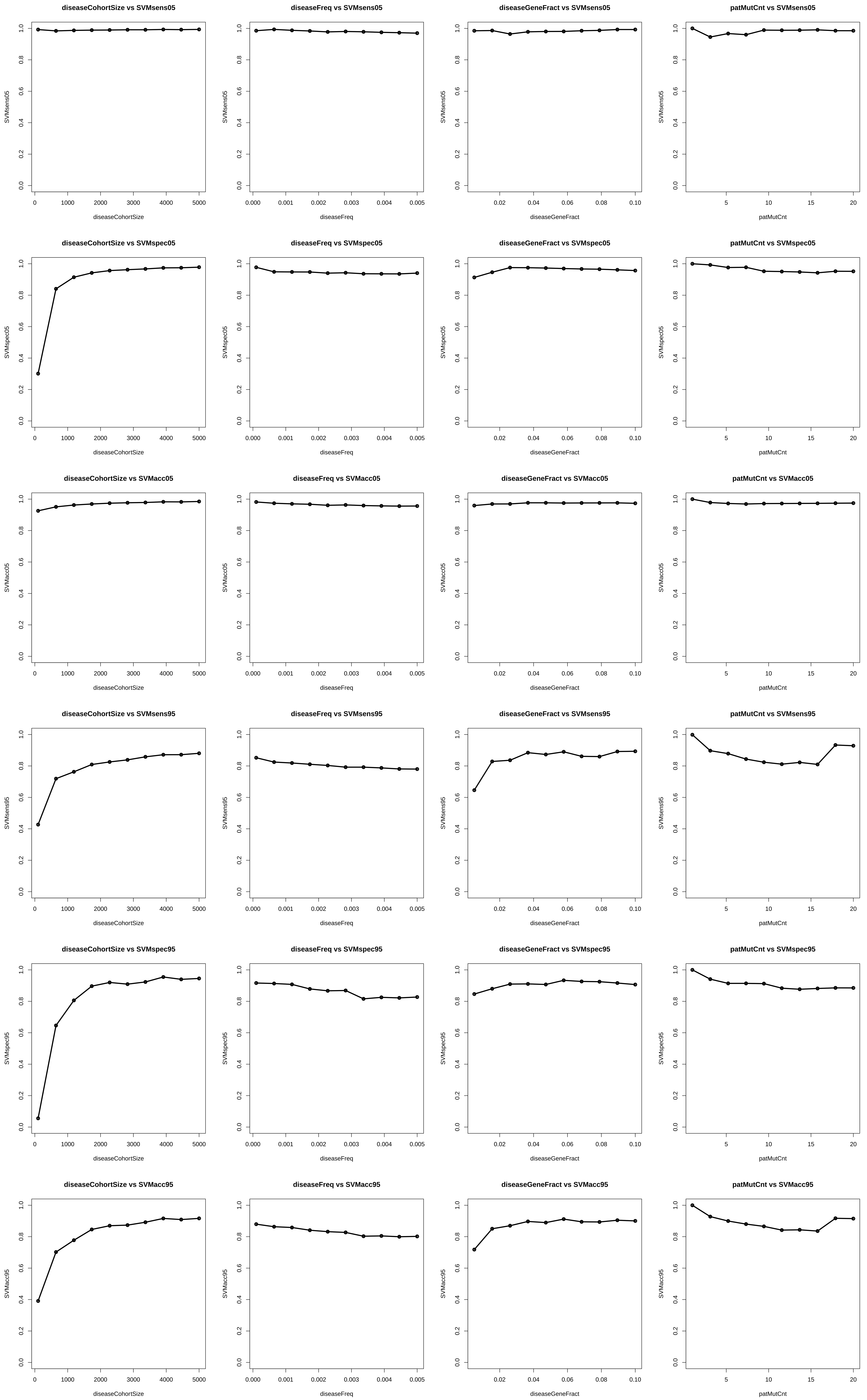

Supplementary Figure S2: Sensitivity of variant classification to input parameters (continue)

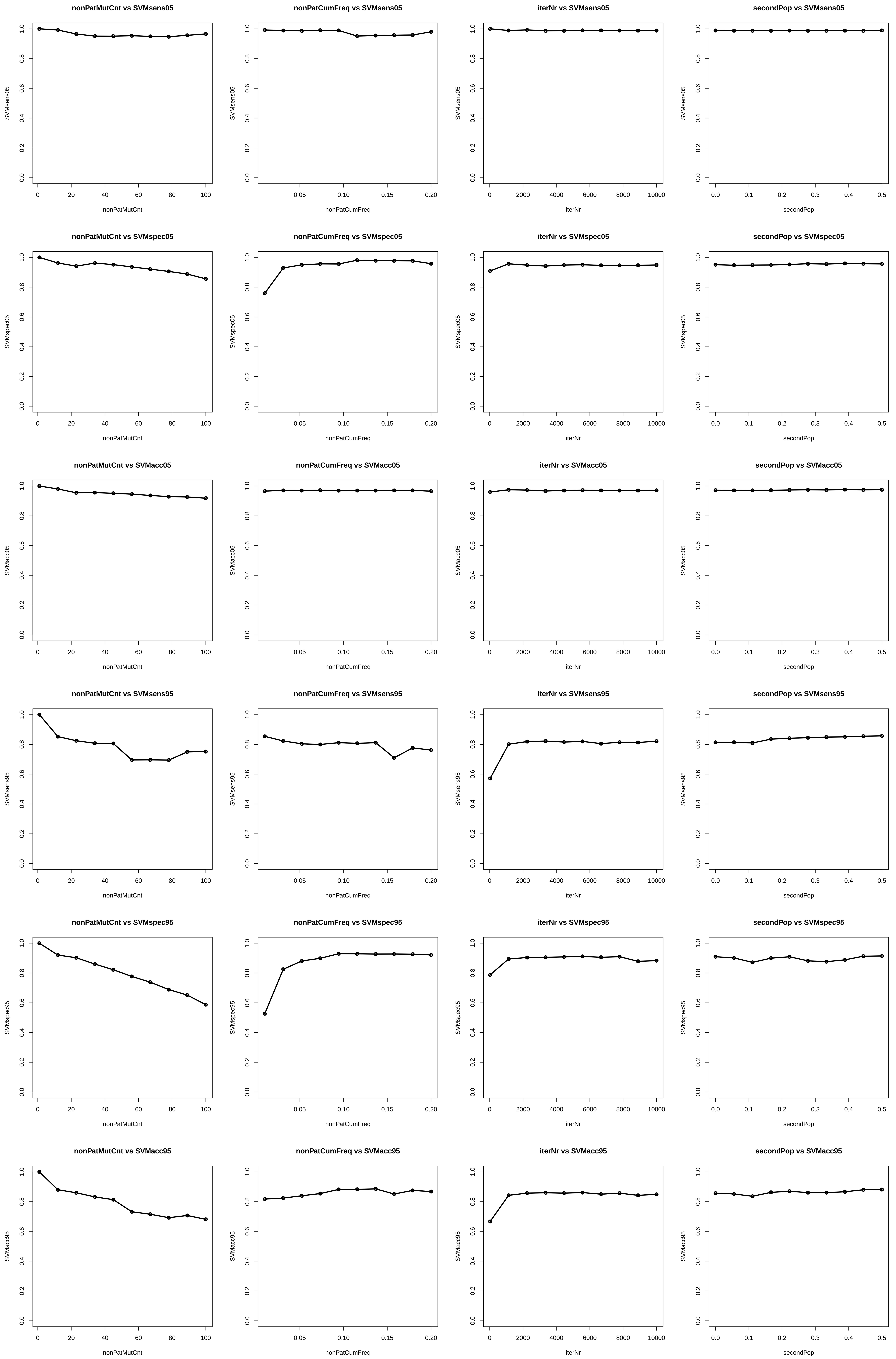

Abbreviations of X labels: diseaseCohortSize − disease cohort size (default=2000); diseaseFreq − frequency of disease individuals within the population (default=0.001); diseaseGeneFract − the fraction of disease individuals affected by bi−allelic variants in the GENE (default=0.02); patMutCnt − the number of distinct pathogenic mutations (default=10); nonPatMutCnt − the number of distinct non−pathogenic (default=20); nonPatCumFreq − cumulative frequency of non−pathogenic mutations (default=0.05); iterNr − number of repetitions of ISVS experiments (default=10000); secondPop − the ratio of population sizes (default=0, i.e. single population)

Abbreviations of Y labels: SVMsens05 − the sensitivity of variant classification with condidence > 50%; SVMspec05 − the specificity of variant classification with condidence > 50%; SVMacc05 − the accuracy of variant classification with condidence > 50%; SVMsens95 − the sensitivity of variant classification with condidence > 95%; SVMspec95 − the specificity of variant classification with condidence > 95%; SVMacc95 − the accuracy of variant classification with condidence > 95%

Supplementary Figure S3: Sensitivity of ISVS and variant classification to the number and cumulative frequency of non−pathogenic mutations

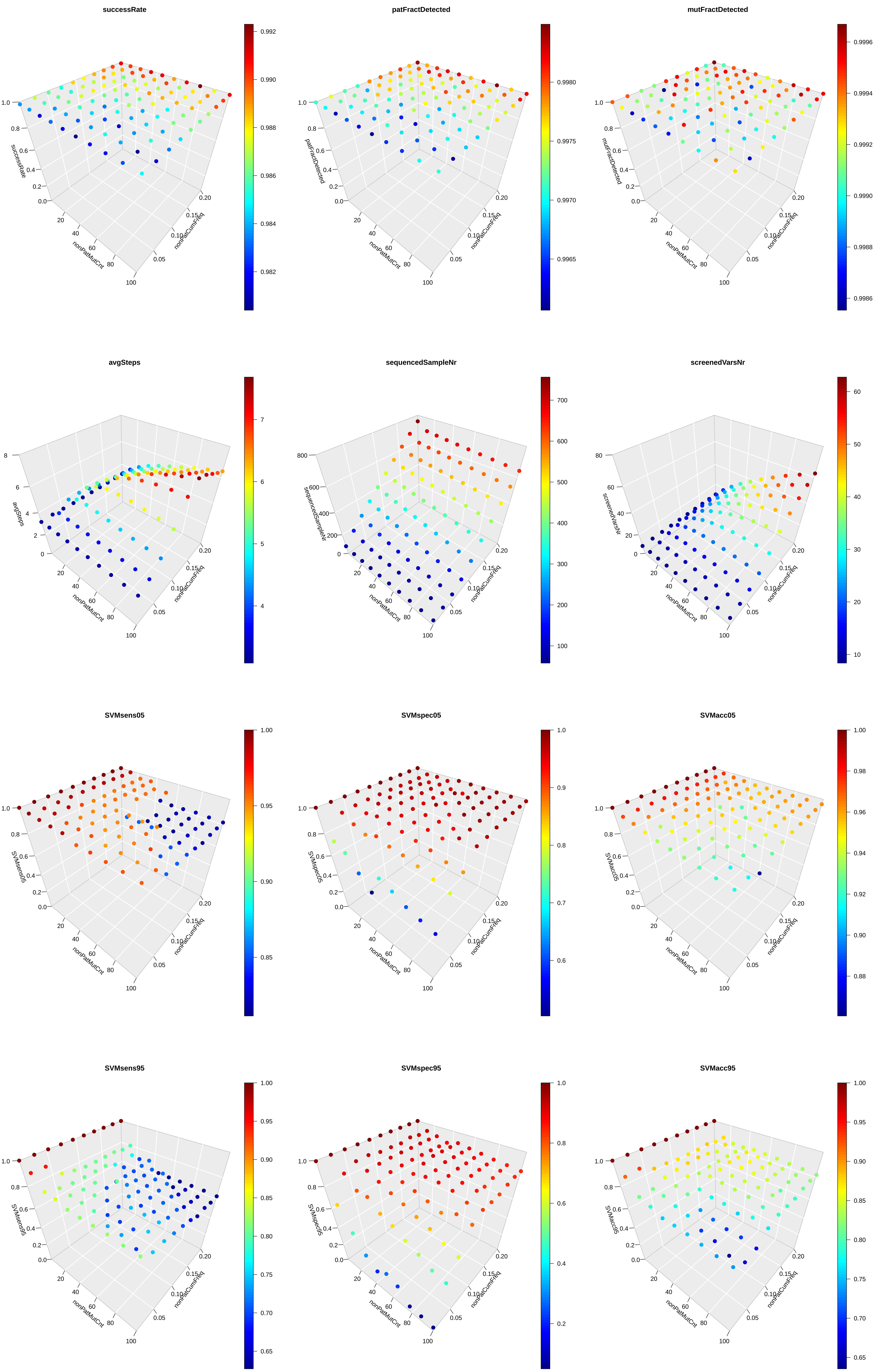

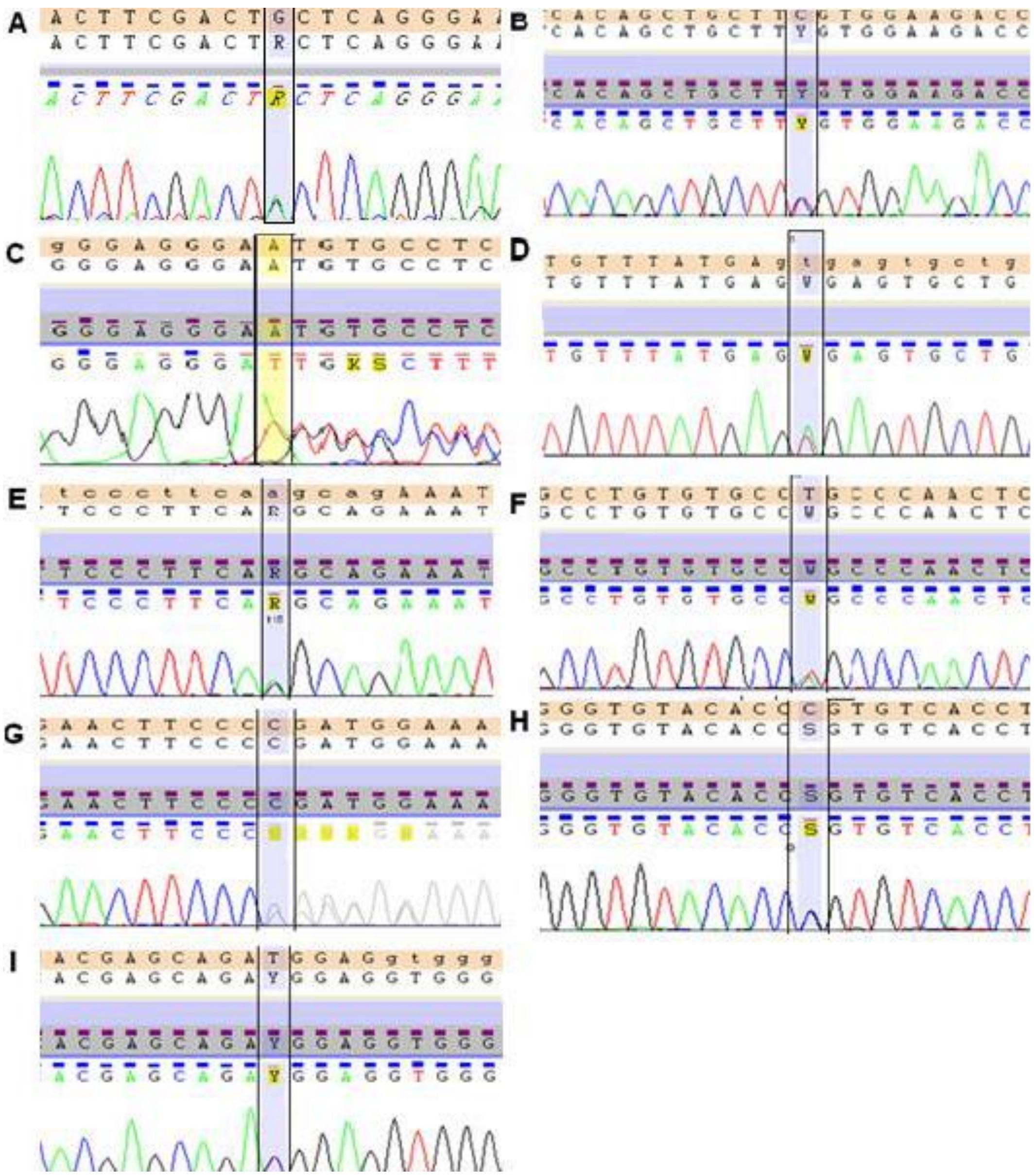

Supplementary figure S4. Sanger sequencing chromatograms showing novel variants identified in the *TMPRSS3* gene: (A) c.218G>A (p.C73Y), (B) c.371C>T (p.S124L), (C) c.579dupA (p.C194Mfs*17), (D) c.782+2T>A, (E) c.953-5A>G (F) c.974 T>A (G) c.999delC (p.D334Mfs*24), (H) c.1306C>G (p.R436G), (I) c.1343 T>C (p.M448T).

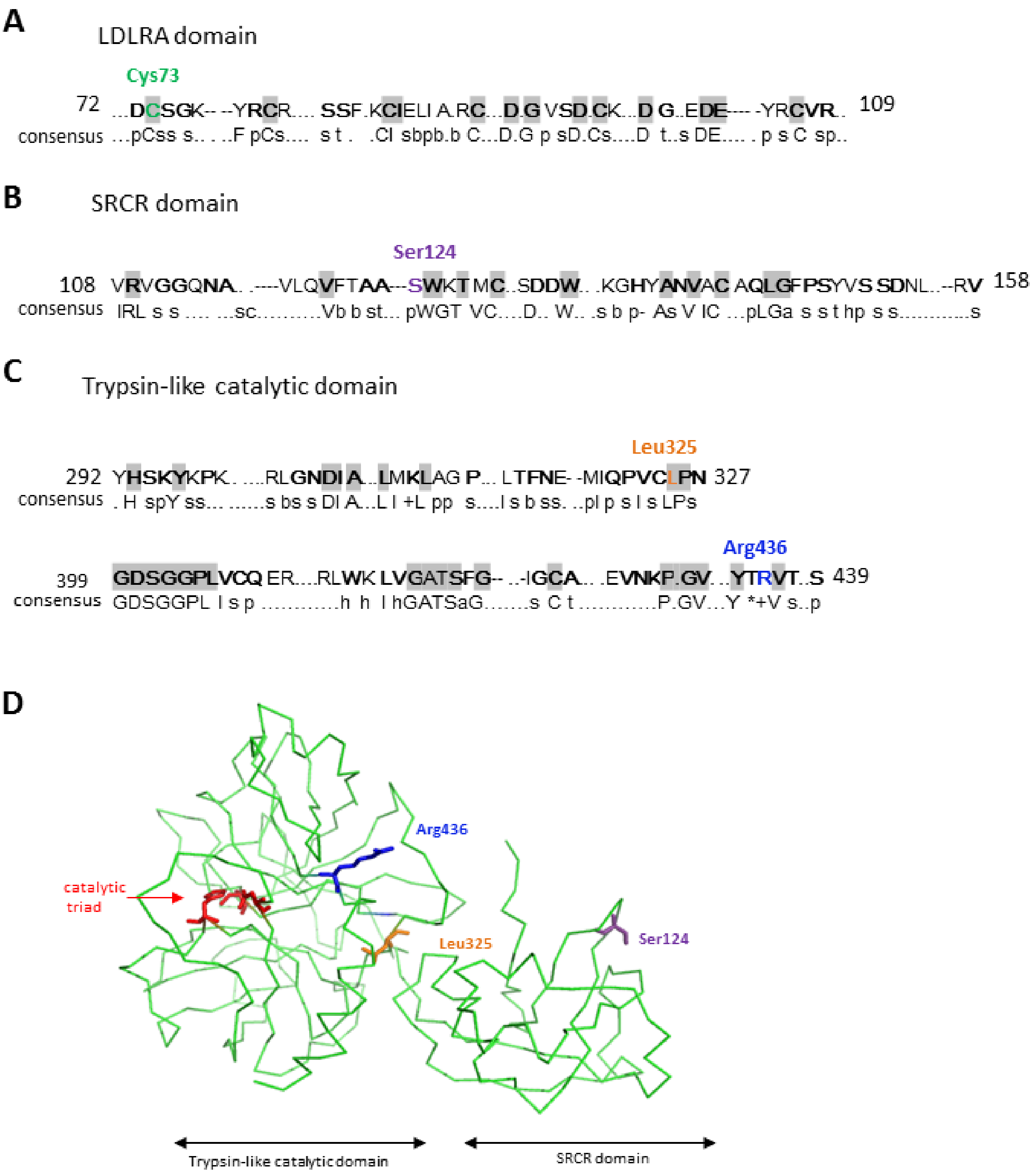

Supplementary figure S5. Conservation of mutated TMPRSS3 variants within LDLRA (**A**), SRCR (**B**), and Trypsin-like catalytic (**C**) domains. The corresponding TMPRSS3 sequences were aligned to SMART domain consensus (60%). Conserved residues are given a grey background. Codes used in a consensus are the following: -; negatively charged (D, E), *; (S, T), l; aliphatic (I, L, V), +; positive (H, K, R), t; tiny (A, G, S), a; aromatic (F, H, W, Y), c; charged (D, E, H, K, R), s; small (A, C, D, G, N, P, S, T, V), p; polar (C,D,E,H,K,N,Q,R,S,T), b; big (E,F,H,I,K,L,M,Q,R,W,Y), h; hydrophobic (A,C,F,G,H,I,L,M,T,V,W,Y). **(D)** –Model of TMPRSS3 comprising two domains, SRCR and catalytic, displayed as ribbons, with mutated residues and active site catalytic triad (colored red) shown in stick representations.

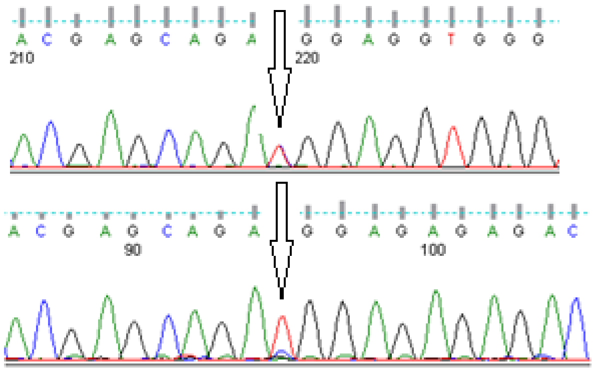

Supplementary figure S6. Chromatograms of g.DNA (A) and c.DNA (B) for carrier of p.M448T mutation in *TMPRSS3* gene.

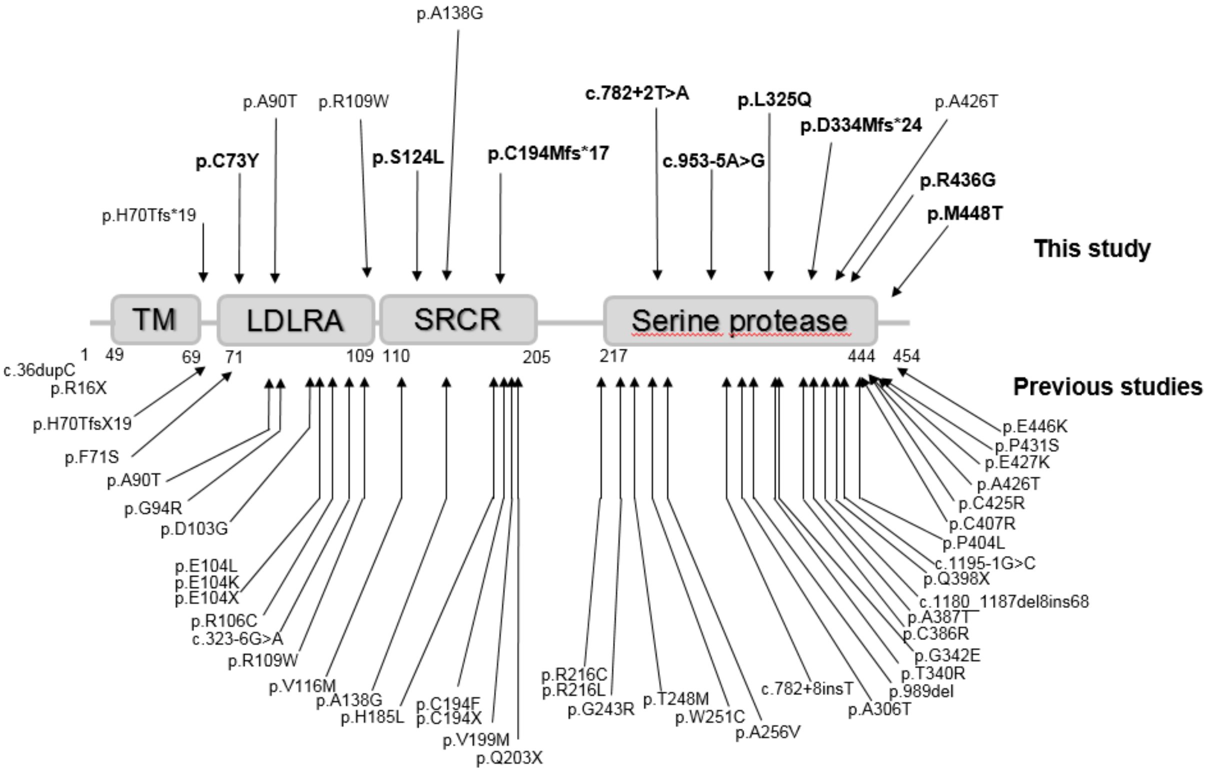

Supplementary figure S7. Localization of novel TMPRSS3 variants relative to domains of the protein and previously reported mutations. Novel mutations are written in bold.
